# Etymologia: *Batrachochytrium salamandrivorans*

**DOI:** 10.3201/eid2207.ET2207

**Published:** 2016-07

**Keywords:** etymologia, Batrachochytrium salamandrivorans, amphibians, salamanders, frogs, fungi, fungal infection, skin destruction

## *Batrachochytrium salamandrivorans* [bə-trayʹ-koh-kitʺ-ri-um saʺ-la-man-dri-vo’rans] 

*Batrachochytrium salamandrivorans* (Figure) is a recently discovered fungus that kills amphibians. It is related to *B. dendrobatidis,* which also kills amphibians (from the Greek *dendron*, “tree,” and *bates*, “one who climbs,” referring to a genus of poison dart frogs). *Batrachochytrium* is derived from the Greek words *batrachos*, “frog,” and *chytra,* “earthen pot” (describing the structure that contains unreleased zoospores); *salamandrivorans* is from the Greek *salamandra*, “salamander,” and Latin *vorans,* “eating,” which refers to extensive skin destruction and rapid death in infected salamanders.

**Figure:**
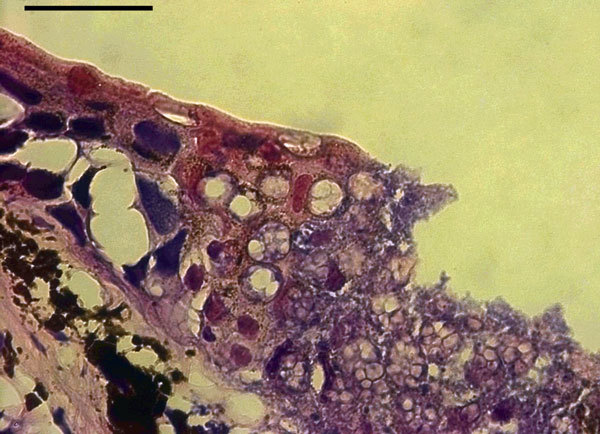
Basal infection in skin of a fire salamander (Salamandra salamandra) characterized by extensive epidermal necrosis, high numbers of intra-epithelial colonial chytrid thalli, and loss of epithelial integrity. Photo by A. Martel and F. Pasmans, courtesy Wikipedia.
